# Integrated analysis of endoplasmic reticulum stress regulators’ expression identifies distinct subtypes of autism spectrum disorder

**DOI:** 10.3389/fpsyt.2023.1136154

**Published:** 2023-04-17

**Authors:** Yanjun Li, Songyin Gao, Yuelan Meng

**Affiliations:** Department of Psychiatry, Zhumadian Second People's Hospital, Zhumadian, China

**Keywords:** autism spectrum disorder, endoplasmic reticulum stress, weighted gene co-expression network analysis, machine learning, consensus clustering analysis, immune microenvironment

## Abstract

Endoplasmic reticulum (ER) stress has been demonstrated to play important roles in a variety of human diseases. However, their relevance to autism spectrum disorder (ASD) remains largely unknown. Herein, we aimed to investigate the expression patterns and potential roles of the ER stress regulators in ASD. The ASD expression profiles GSE111176 and GSE77103 were compiled from the Gene Expression Omnibus (GEO) database. ER stress score determined by the single sample gene set enrichment analysis (ssGSEA) was significantly higher in ASD patients. Differential analysis revealed that there were 37 ER stress regulators dysregulated in ASD. Based on their expression profile, the random forest and artificial neuron network techniques were applied to build a classifier that can effectively distinguish ASD from control samples among independent datasets. Weighted gene co-expression network analysis (WGCNA) screened out the turquoise module with 774 genes was closely related to the ER stress score. Through the overlapping results of the turquoise module and differential expression ER stress genes, hub regulators were gathered. The TF/miRNA-hub gene interaction networks were created. Furthermore, the consensus clustering algorithm was performed to cluster the ASD patients, and there were two ASD subclusters. Each subcluster has unique expression profiles, biological functions, and immunological characteristics. In ASD subcluster 1, the FAS pathway was more enriched, while subcluster 2 had a higher level of plasma cell infiltration as well as the BCR signaling pathway and interleukin receptor reaction reactivity. Finally, the Connectivity map (CMap) database was used to find prospective compounds that target various ASD subclusters. A total of 136 compounds were significantly enriched. In addition to some specific drugs which can effectively reverse the differential gene expression of each subcluster, we found that the PKC inhibitor BRD-K09991945 that targets Glycogen synthase kinase 3β (GSK3B) might have a therapeutic effect on both ASD subtypes that worth of the experimental validation. Our finding proved that ER stress plays a crucial role in the diversity and complexity of ASD, which may inform both mechanistic and therapeutic assessments of the disorder.

## 1. Introduction

Autism spectrum disorder (ASD) is a complex, early-onset neurodevelopmental disorder. Its core symptoms are social interaction deficits, communication impairments, and repetitive stereotyped behaviors (1). According to the Centers for Disease Control and Prevention, ASD affects roughly 1 in every 54 children (2), and the prevalence is rising over time. Patients with ASD sometimes have co-morbid conditions like intellectual disabilities, sleep disorders, and schizophrenia (3–5), which places a significant cost on families and society. Although the etiology and pathogenesis of autism are currently unknown, it is generally believed that risk factors for ASD include anomalies in neurodevelopment (6, 7), neuroinflammation (8), oxidative stress (9), viral infections in the mother (10), and alterations in gut microbes (4). Until now, the diagnosis of ASD still mainly relied on subjective methods such as parental interviews and symptom scales. However, early ASD diagnosis and treatment have a direct impact on the recovery and development of ASD-affected youngsters (11), therefore, a deeper understanding of the mechanism underlying ASD is critical to the development of more accurate diagnoses and effective treatments.

In eukaryotic cells, the endoplasmic reticulum is a reticular organelle made up of many tubular structures and flattened vesicles. It is primarily in charge of protein synthesis, processing, and transport, as well as lipid and sterol biosynthesis. When cells are driven by a variety of physicochemical variables (such as hypoxia, aberrant Ca2+ concentration, hunger, viral infection, etc.), the normal folding and modification of proteins are hampered, and the unfolded protein response (UPR) is activated, leading to the accumulation of misfolded proteins in the ER (12). In this case, IRE1 (inositol-requiring protein 1), PERK (PKR [double-stranded-RNA-dependent protein kinase]-like ER kinase), and ATF6 (activating transcription factor 6), regarded as sensors of ER stress, initiate the intracellular signaling pathways to promote the in-folding or degradation of a protein, thus maintaining cellular homeostasis (13–15). However, apoptosis is produced when cells are subjected to high amounts of ER stress for an extended period (16).

Recent research has revealed that endoplasmic reticulum (ER) stress, a protective stress response, has emerged to play a significant role in many diseases and may present new prospects for ASD. Amanda Crider and his colleague found significant increases in the mRNA levels of ATF4, ATF6, PERK, XBP1, CHOP, and IRE1 in the middle frontal gyrus of ASD subjects, and this change was positively connected with the diagnostic score of ASD stereotypic behavior (17). Further, Daoyin Dong and his team compared the activation of ER stress signals in different brain regions and they found that IRE1α was activated in the cerebellum and prefrontal cortex but ATF6 was activated in the hippocampus (18). In particular, genetic variations in several synaptic genes (such as GPR85 (19), NLGN3 (20), and CADM1 (21)) implicated in ASD have been shown to induce ER stress genes. These findings are intriguing, but they only focus on the expression of a small fraction of ER stress molecules in ASD, without exploring the possible mechanisms involved and the heterogeneity of ASD. Therefore, a thorough analysis of the various ER stress regulators expression profiles between normal tissues and ASD, the distinct subtypes, as well as the immunological features of ASD would help supply new ideas for clinical prevention and precision treatment.

In the present study, we systematically evaluate the expression pattern of the ER stress regulators in ASD. We found that ER stress regulators can well distinguish between control and ASD patients. WGCNA analysis revealed that the turquoise module was closely linked to the elevated ER stress scores in ASD. Furthermore, we clustered ASD samples based on the core ER stress regulators. Each subtype has distinct expression profiles, biological functions and immunological characteristics. Besides, we anticipated compounds for different subclusters to achieve accurate treatment, and the PKC inhibitor BRD-K09991945, which targets GSK3B, may have a positive therapeutic impact on both subclusters and is therefore worth further investigation.

## 2. Methods

### 2.1. Data collecting and pre-processing

We collected the expression profile datasets GSE111176 (22, 23), GSE77103 (24) and GSE38322 (25) of ASD from the GEO database by R package “GEOquery” (26). The dataset GSE111176 was contributed by Vahid H Gazestani et al., which contains information on the expression profiles of leukocytes derived from 119 ASD patients and 126 controls. One validation set GSE77103, which includes the expression patterns of peripheral blood mononuclear cells obtained from 4 healthy controls and 4 ASD patients, was submitted by Inoue R et al. Another validation set GSE38322, contains the expression patterns of brain tissues sourced from 18 controls and 18 ASD patients, was contributed by the Matthew Ginsberg et al. All samples were included in this analysis. The GPL10558 (Illumina HumanHT-12 V4.0 expression bead chip) was used for the GSE11176 dataset and the GSE38322 and the GPL17077 (Agilent-039494 SurePrint G3 Human GE v2 8x60K Microarray 039381 (Probe Name version)) was used for the GSE77103 dataset. During data processing, the “removeBatchEffect” function of the “limma” (27) package was used to remove the batch effect of the sub-datasets of the GSE111176. And the expression profiles of three datasets were normalized by the “normalizeBetweenArrays” function of the “limma” package. Finally, Gene probes were annotated with official gene symbol, and mean values were taken if multiple gene probes matched to the same gene.

### 2.2. Collection of the reticulum stress-related genes

From the Molecular Signatures Database V7.0 (MSigDB) (28), we retrieved the endoplasmic reticulum stress-related gene sets “GOBP NEGATIVE REGULATION OF RESPONSE TO ENDOPLASMIC RETICULUM STRESS” and “GOBP POSITIVE REGULATION OF RESPONSE TO ENDOPLASMIC RETICULUM STRESS,” which includes a total of 256 genes.

### 2.3. Differential gene expression analysis

The “limma” package of the R software was applied for differential gene analysis. The screening criteria for the DEGs of normal and ASD patients were |logFC| > 0 and an adjusted value of *p*<0.05. As for the ASD patterns, the threshold was set as |logFC| > 0.2 and pvalue<0.05. The R packages “ComplexHeatmap” (29) and “ggplot2” (30) were used to plot the findings of the differential analysis. The R package “RCircos” (31) is used to display information on the chromosomal location of differential genes.

### 2.4. Machine learning methods

Two machine learning (random forest (RF) and artificial neural network (ANN)) approaches were applied to construct the ASD classifier. Random forest regression is a machine-learning algorithm that takes an ensemble learning approach for prediction. It is made up of various decision trees, each trained on random features. ANN consists of the input layer, the hidden layer and the output layer. The number of neurons in the input layer indicates the number of features being evaluated; while the neurons in the output layer are the dependent variables; Each neuron in the hidden and output layers is linked to all neurons in the previous layer by the corresponding numerical weights. To develop the predictive signature, the differentially expressed endoplasmic reticulum stress genes were subjected to random forest analysis using the R package “randomForest” (32), and genes with gene importance scores greater than 4 were selected for the further neural network created using the “NeuralNetTools” (33) and “neuralnet” packages (34). Using the R package pROC (35), the ROC curve was drawn and the area under the curve was determined to assess the distinguishing performance of the signature. Additionally, the external validation set GSE77103 confirms the classifier’s diagnostic effectiveness.

### 2.5. WGCNA

Weighted Gene Correlation Network Analysis (WGCNA) (36) is mainly used to identify co-expressed gene modules and examine the connection between gene networks and phenotypes. For computational efficiency, the top 25% of variant genes based on an analysis of variance were selected for WGCNA analysis using the “WGCNA” package (36). The soft threshold was calculated by the “picksoftthreshold” function and scale-free networks were built. Subsequently, the adjacency matrix was transformed into a topological overlap matrix (TOM). The hierarchical clustering and dynamic tree-cut method were used to identify gene modules. Co-expression modules were defined using a minimum module size of 50 genes and by merging modules with a module eigengene dissimilarity below 0.2. Finally, the correlations between modules and clinical features were determined by spearman correlation analysis.

### 2.6. Identification of the ER stress patterns

The varied ASD patterns were identified using the ConsensusClusterPlus (37) R package, and the clustering was carried out using the pam method with a sampling proportion of 0.8, and it was employed for 100 iterations to assure clustering stability. The clustering score for the cumulative distribution function (CDF) curve was calculated to estimate the ideal number of clustering. The consensus clustering’s reliability was confirmed by Principal Component Analysis (PCA).

### 2.7. Biological enrichment analysis for the different ER stress patterns

Based on the differentially expressed genes, Gene Ontology (GO) (38) terms, which provide context for cellular component (CC), molecular function (MF), and biological process (BP), as well as the Kyoto Encylopaedia of Genes and Genomes (KEGG) analyses (39), were employed to assess the biological changes of ASD patterns. We used the R package “clusterprofiler” (40) for GO and KEGG analysis and the R package “pathview” (41) to visualize the significantly distinct KEGG pathways between ASD patterns. Terms with a false discovery rate (FDR) < 0.05 were considered statistically significant. Gene Set Enrichment Analysis (GSEA) (42) is a method that determines whether *a priori*-defined set of genes show statistically significant expression variations between two biological states. From the MSigDB database, we have gathered the gene set “c2 cp all v7.0 symbols.gmt.” The GSEA analysis was performed using the R package “clusterprofiler” and it was determined that the |normalized enrichment score| (|NES|) > 1 and FDR < 0.25 were significant results.

### 2.8. Master regulator analyses

Master regulator analysis (MRA) (43) is an algorithm used to infer transcription factors (TFs) controlling the transition between the two phenotypes and the maintenance of the latter phenotype. In this study, we applied a recently developed coexpression-based gene network inference and interrogation tool, corto (44), to determine the master regulator genes of the transition from normal control to ASD subtypes. The collection of human transcription factors was used as the potential centroids for the model input, along with the gene expression matrix of normal controls and two ASD subclusters. And then, a list of master regulators sorted by normalized enrichment scores (NES) was obtained through the “mra” function of “corto” package. The NES is positive if the centroid network is higher in the sample vs. the mean of the dataset, negative if lower.

### 2.9. Correlation analysis between ER stress patterns and immune characteristics

The analytical tool CIBERSORTx (45), developed by Newman et al., uses gene expression data to perform cell-type deconvolution and offers an estimate of the abundances of member cell types in a mixed cell population. The online tool CIBERSORTX (https://cibersortx.stanford.edu/) was applied to estimate immune cell infiltration in ASD patterns. Single sample gene set enrichment analysis (ssGSEA) was performed according to previous studies, which was used to determine enrichment scores (ES) for each coupling of a sample and immune reaction gene sets in the ImmPort database.^1^ The R package “GSVA” (46)was used for the ssGSEA analysis and “ggplot2” for visualization. The Wilcox test was used to assess the enrichment scores representing immunocyte abundance and immune response activity between ASD patterns.

### 2.10. miRNA-gene and TF-gene interaction network

NetworkAnalyst^2^ (47) is an online visualization and analysis platform which can perform differential gene expression analysis, protein–protein interaction analysis and integrated analysis of multiple datasets. With the use of this platform, we were able to obtain chromatin immunoprecipitation data from the ENCODE database (48) for the construction of TF-gene network and experimentally verified data for the construction of miRNA-gene interaction network from the miRTarBase database (49). Afterward, the Cytoscape software (50) was utilized for visualization.

### 2.11. Identification of the candidate compounds for the ER stress patterns

The Connectivity Map database (Cmap) (51) focuses on the interaction between compounds, genes and disease states. It contains induced gene expression profiles of more than 25,200 perturbagens of which ~19,800 are small molecules, 314 biologics and ~ 5,075 genes with altered function by shRNA, cDNA, and/or CRISPR. These perturbagens were assayed in different cell lines to produce around 1.3 million individual gene expression profiles corresponding to ~473,000 gene expression signatures (52). Each ASD subcluster’s differentially expressed genes were submitted to the Camp database, and the connectivity score—which measures how closely compounds-induced transcript modifications resemble user-input differential gene alterations—was obtained. Additionally, the mechanism of action (MoA) of the compounds was analyzed.

### 2.12. Statistical analysis

R programming (version 4.0.2, available at https://www.rproject.org) was used for all data calculations and statistical analysis. The Spearman test was utilized for correlation analysis. *p* < 0.05 was considered statistically significant.

## 3. Results

### 3.1. The landscape of ERS regulators between control and ASD samples

**Figure 1:**
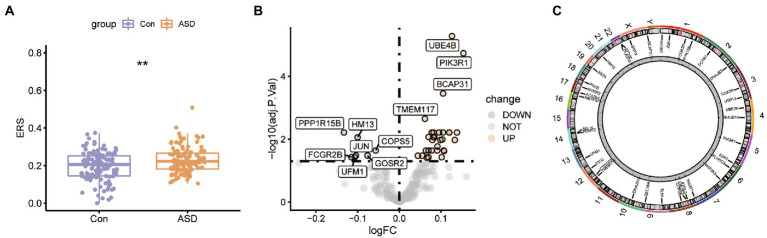
The expression pattern of the ER stress regulators in ASD. **(A)** Box plot for ER stress score between control ASD group. ^*^p < 0.05, ^**^p < 0.01. **(B)** The volcano plot of the 256 ER stress regulators in control and ASD groups. **(C)** Chromosomal positions of the differential expressed ER stress regulators.

The ASD expression profiles were downloaded from the GEO database, and the mean values of gene expression for each sample were essentially the same after normalization (Supplementary Figure S1). We have estimated the ER stress score for each sample in the dataset using the ssGSEA method. The Wilcox test results highlighted the significant upregulation in the ER stress score between the ASD and normal samples (Figure 1A), which indicates that ER stress has an important role in the development and progression of ASD. Further, the expression of the ER stress genes was evaluated in the two groups, and 37 genes with differential expression were found, including 30 up-regulated genes and 7 down-regulated genes (Figure 1B). Figure 1C displays the chromosomal locations of the ER stress genes that are differentially expressed.

### 3.2. Construction of the ASD classifier and validation

**Figure 2:**
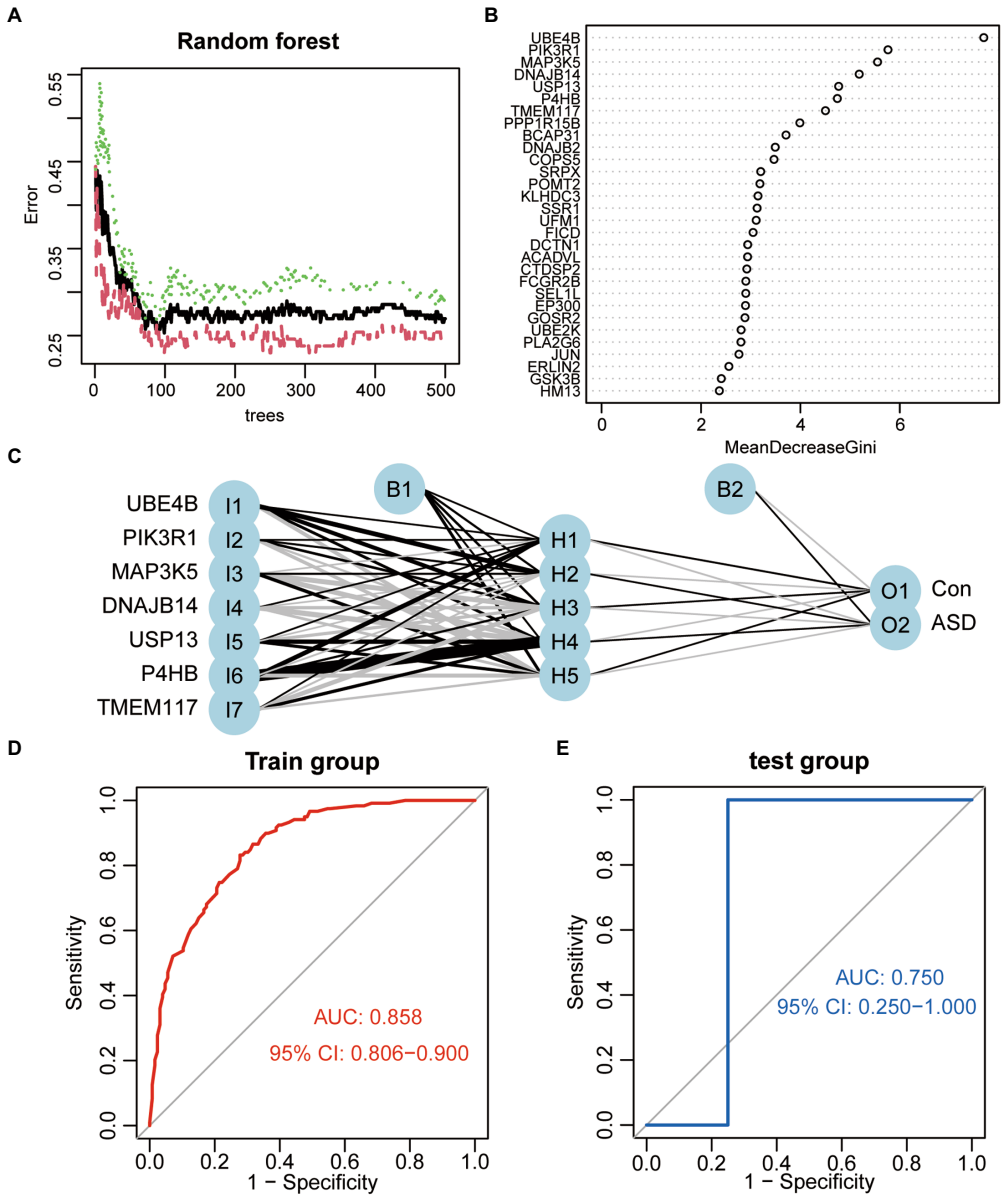
Construction of the ASD classifier. **(A)** The average error rate of random forest model. **(B)** Variable importance ordered by the gini index of a mean decrease in random forest. **(C)** The schematic diagram of artificial neural network model. **(D)** ROC curves show the AUC values of the ASD classifier in GSE111176. **(E)** ROC curves show the AUC values of the ASD classifier in GSE77103. **(F)** ROC curves show the AUC values of the ASD classifier in GSE38322.

To investigate differentially expressed ER stress regulators’ contribution to the diagnosis of ASD, a series of bioinformatic algorithms were employed. We used a random forest to filter the variables, which showed the lowest average error rate at 99 trees (Figure 2A), and then we analyzed the importance of the variables by calculating the Gini index of a mean decrease (Figure 2B). Seven variables with the mean decrease Gini index greater than 4 were selected for subsequent analysis, and they were UBE4B, PIK3R1, MAP3K5, DNAJB14, USP13, P4HB, TMEM117, respectively. The above-mentioned seven genes were used as input variables into the artificial neural network, a five-neuron layer that extracts and fuses features from the input layer was used to determine the final binary classification result using well-trained weight coefficients and bias (Figure 2C). ROC analysis was implemented to determine the value of the classifier in diagnosing ASD, the results revealed that this classifier was effective at differentiating between healthy people and ASD patients (Figure 2D). Additionally, we verified its effectiveness and dependability in two independent validation set GSE77103 and GSE38322, both of which had AUC value of 0.75 (Figure 2E) and 0.759 (Figure 2F), respectively.

### 3.3. Identification of key modules associated with the ERS score

**Figure 3:**
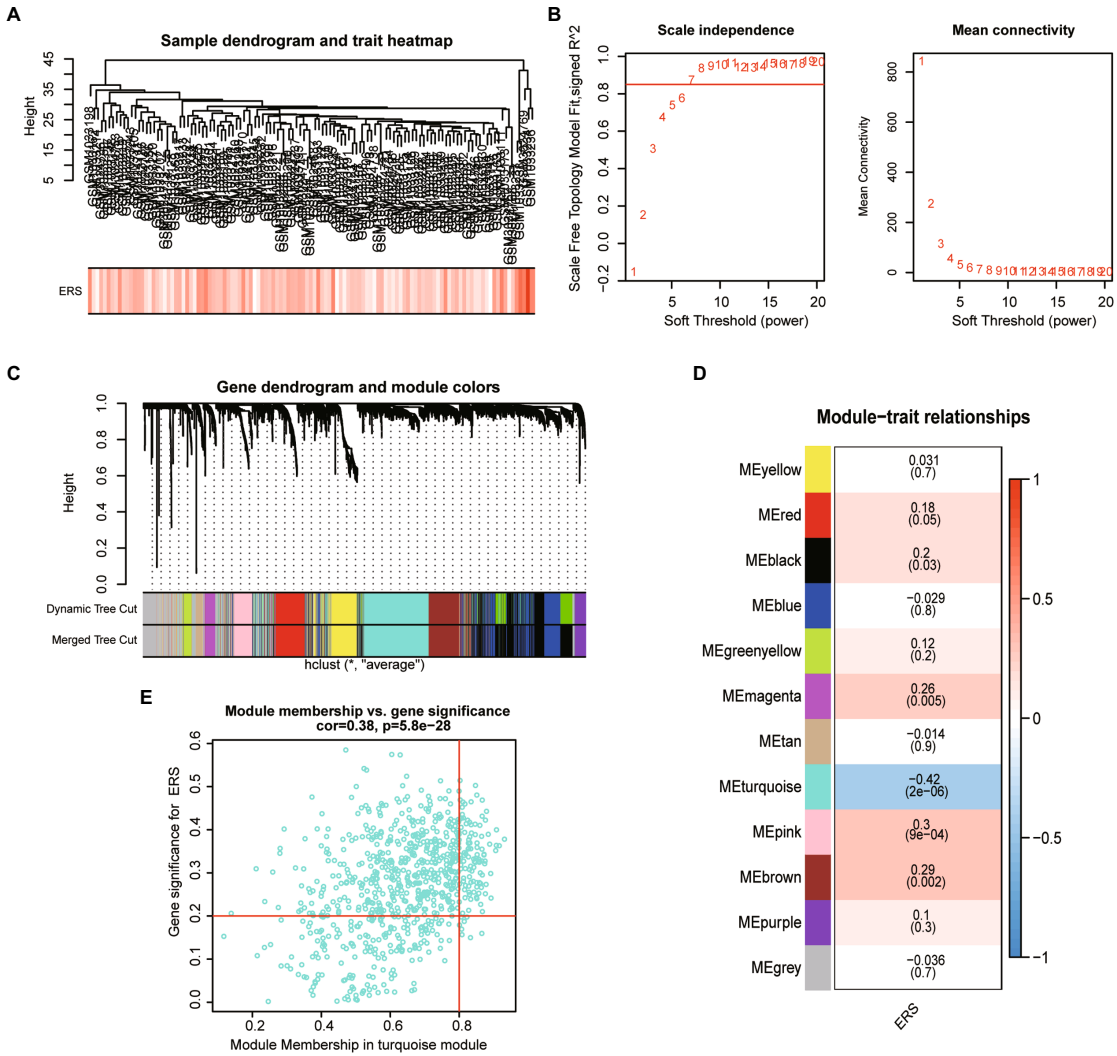
The weighted gene co-expression network analysis. **(A)** Clustering dendrogram of ASD sample with trait heatmap. **(B)** The scale-free topology model fit (*R*^2^) (left) and the mean connectivity of the co-expression network (right) given different soft-thresholding powers. **(C)** Gene dendrogram obtained by average linkage hierarchical clustering. The color row underneath the dendrogram shows the module assignment determined by the Dynamic Tree Cut. **(D)** Heatmap of the correlation between module eigengenes and ER stress score. **(E)** The scatterplot of gene significance (GS) for ER stress score vs. module membership (MM) in the turquoise module.

Given the previous analysis suggested that ER stress may be involved in the development of ASD. We further analyzed the co-expression genes associated with ERS scores in patients with ASD. A dendrogram of ASD patients in GSE11176 with ER stress score was clustered using the average linkage method and Pearson’s correlation method (Figure 3A). To obtain a signed network fulfilling the scale-free topology, the soft-thresholding power parameter was set to 7 (at this point scale-free R2 > 0.85) (Figure 3B). A total of 12 modules that have sizes between 65 and 774 genes were identified in the average hierarchical clustering and dynamic tree clipping (Figure 3C). To find the most correlated modules with ERS scores in ASD, we performed spearman correlation analysis (Figure 3D), and the results showed that the turquoise module appeared to have the highest association with ER stress scores (cor = −0.42, *p* = 2e-06), while magenta (cor = 0.26, *p* = 0.005), pink (cor = 0.3, *p* = 9e-04), brown (cor = 0.29, *p* = 0.002) modules showed a significant positive correlation with ERS scores (Figure 3E). We therefore selected the turquoise module for further analysis, which included 774 genes. A significant correlation existed between the module membership (MM) and gene significance (GS) of the turquoise modules (Figure 3F).

### 3.4. Construction of the miRNA-gene and TF-gene interaction network

**Figure 4:**
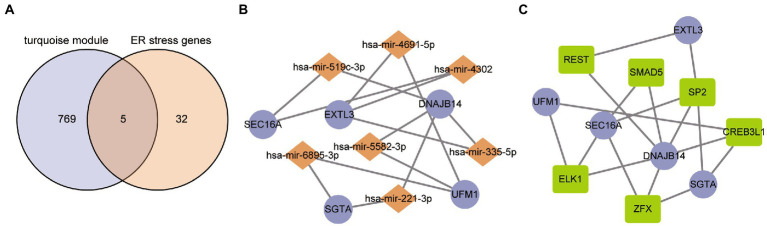
TF/miRNA-hub gene interaction network. **(A)** Hub ER stress regulators representation through a Venn diagram. **(B)** Network for miRNA interaction with hub ER stress regulators. The purple nodes represent the hub ER stress regulators and the other nodes represent miRNAs. **(C)** Network for TF interaction with hub ER stress regulators. The purple nodes represent the hub ER stress regulators and the other nodes represent TFs.

The overlapped results of the turquoise module and differentially expressed ER stress gene was considered to be the hub ER stress genes of ASD. As a result, five genes were obtained, namely SEC16A, EXTL3, DNAJB14, SGTA and UFM1 (Figure 4A). Further, they were subjected to the NetworkAnalyst web to collect the miRNA-gene and TF-gene interactions. The miRNA-gene network consists of 12 nodes and 14 interaction pairs, and DNAJB14 was regulated by 4 miRNAs in this network (Figure 4B). In addition, the TF-gene network includes 11 nodes and 17 interaction pairs (Figure 4C). The transcription factor SP2 may be closely associated with ASD because it regulated four hub ER stress regulators in this network. These 7 miRNAs and 6 TF regulate more than one hub ER stress gene of the network, which indicates high interaction of the miRNA/TF with hub ER stress genes.

### 3.5. ER stress regulators mediated ASD patterns

**Figure 5:**
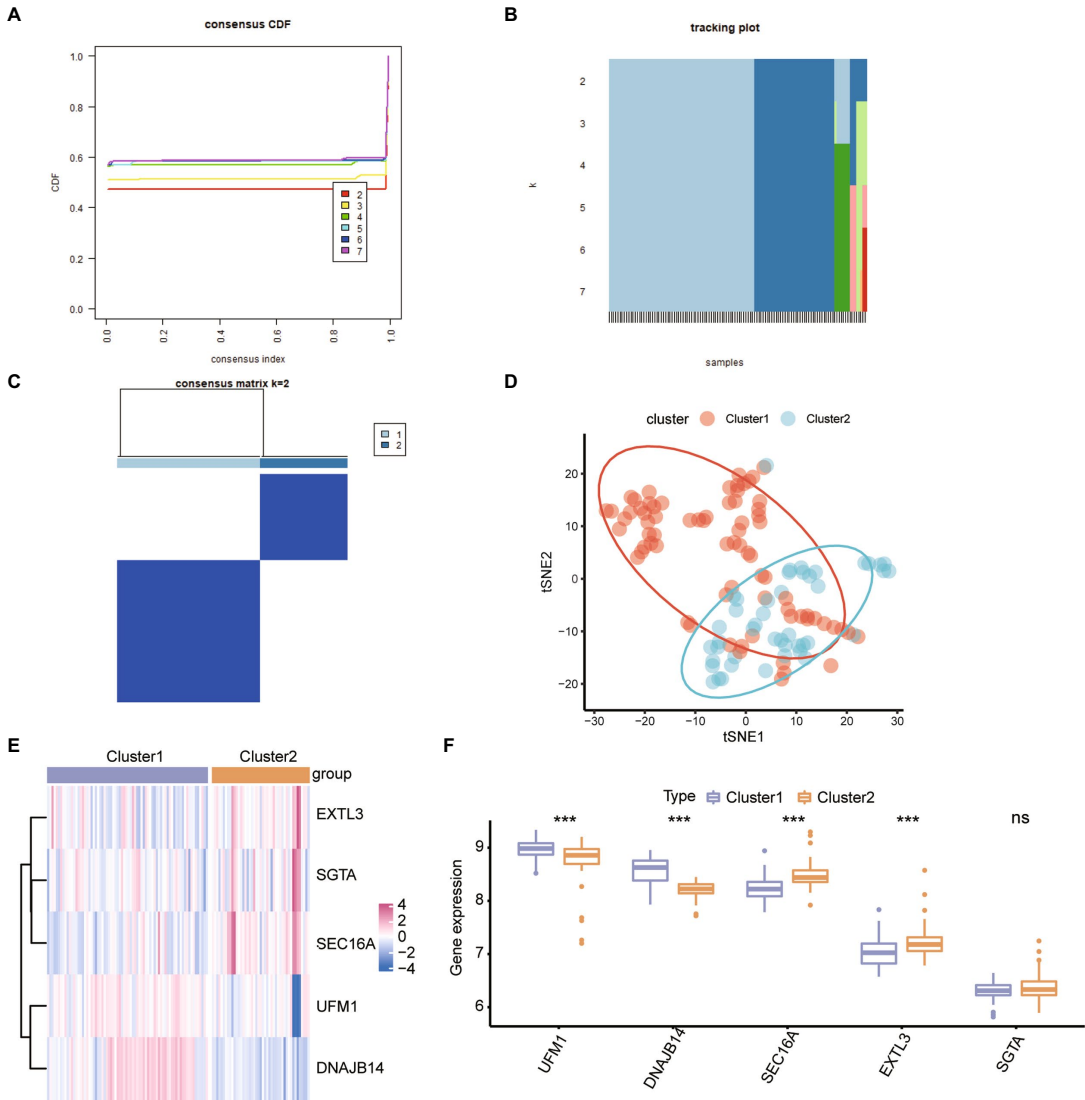
Identification of the ER stress patterns in ASD. **(A)** Consensus clustering cumulative distribution function (CDF) for *k* = 2–7. **(B)** The tracking plot shows the cluster assignment of items (columns) by color for each k (rows). **(C)** Heatmap describing co-occurrence proportions for ASD samples (*k* = 2). **(D)** Tsne analysis for the transcriptome profiles of the ER stress patterns revealed a difference between ASD subclusters. **(E,F)** Heatmap and box plot of hub ER stress regulators’ expression in ASD subclusters. ^*^p < 0.05, ^**^p < 0.01. ^***^ < 0.001.

To study the sub-clusters in ASD, the unsupervised consensus cluster analysis was carried out on ASD patients based on the expression of the hub ER stress regulators. There were found to be two distinct ASD modification subclusters (Figures 5A-C), with 45 samples in cluster 2 and 74 samples in cluster 1. Tsne analysis also verified that ASD patients can be categorized into two patterns (Figure 5D). We also looked at the hub ER stress gene expression levels across the different subtypes, and the results showed that UFM1 and DNAJB14 were up-regulated in subcluster 1, while SEC16A and EXTL3 were up-regulated in subcluster 2 (Figures 5E,F), indicating that the various ASD subclusters may have various transcriptome or other characteristics.

### 3.6. Biological properties and MRA of different ER stress patterns

**Figure 6:**
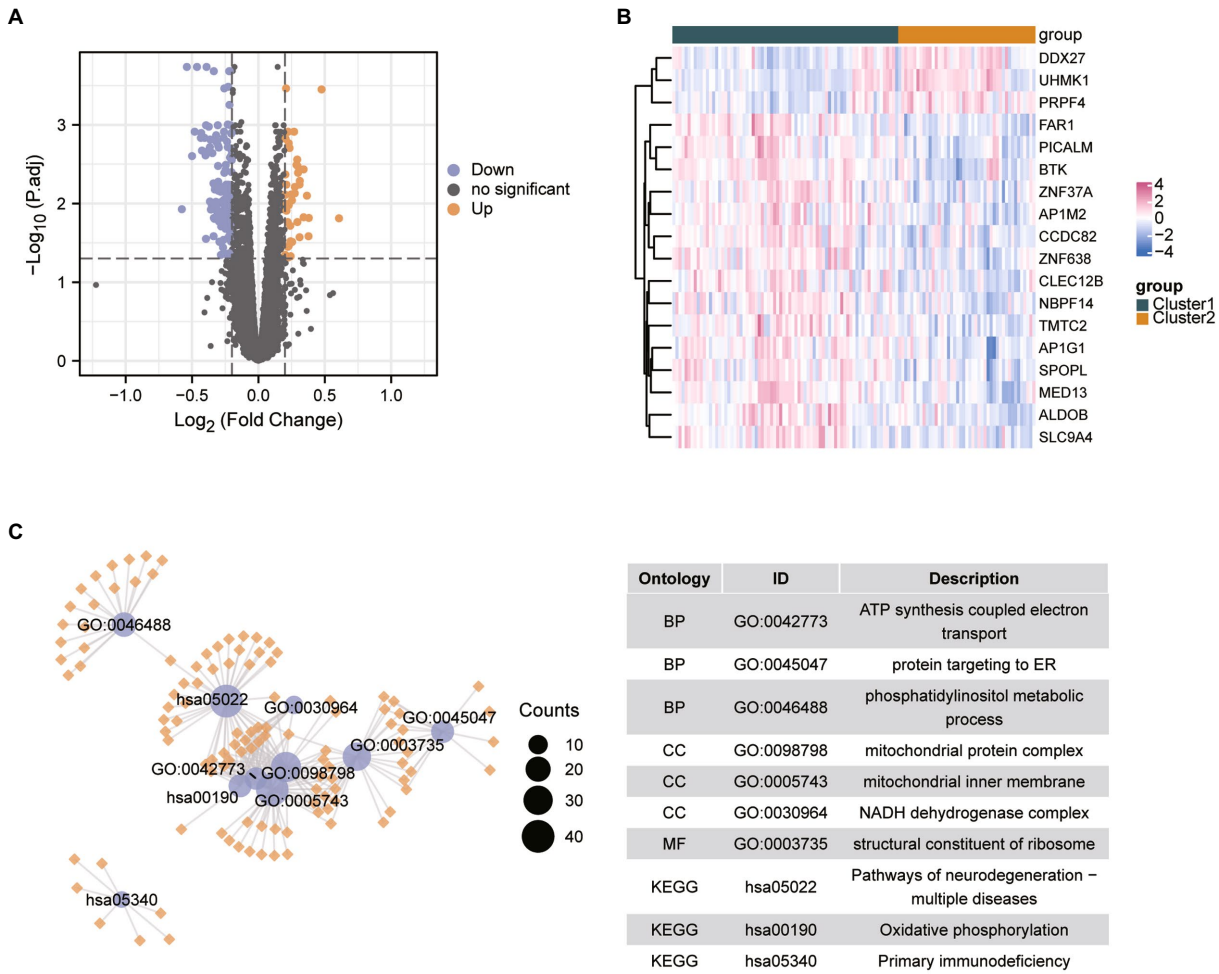
Identification of the differential expressed genes between different clusters in ASD. **(A,B)** Volcano plot and heatmap showed the differential expressed genes in ASD cluster 2 compared with the ASD cluster 1. **(C)** GO and KEGG enrichment analysis based on the differential expressed genes between ASD clusters. Abbreviations: BP, biological processes; CC, cellular components; MF, molecular functions.

**Figure 7:**
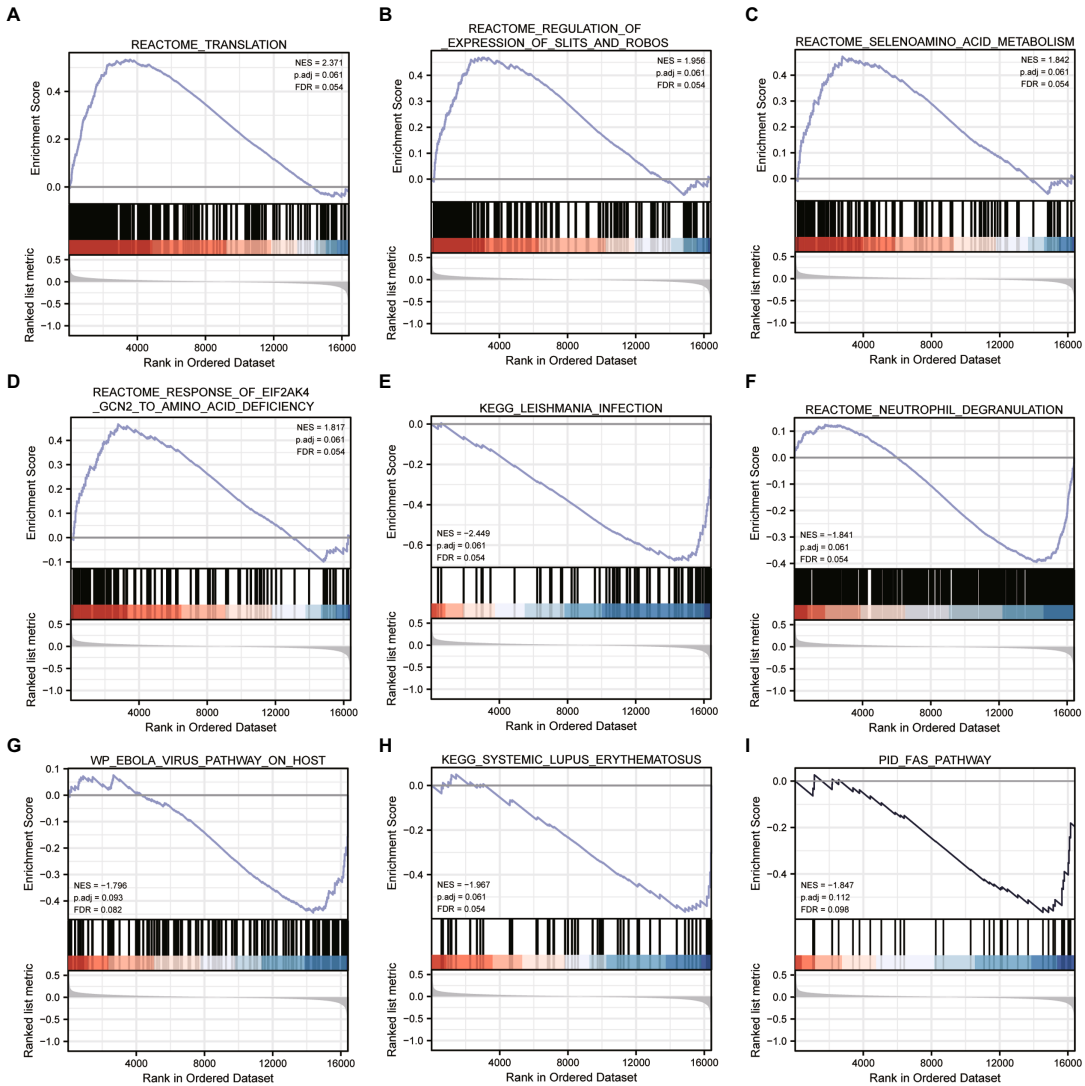
Gene Set Enrichment Analysis between ASD clusters. **(A-I)** Enrichment plots of pathways in c2.cp.all.v7.2.symbols.gn in ASD subcluster 2 obtained after GSEA analysis.

**Figure 8:**
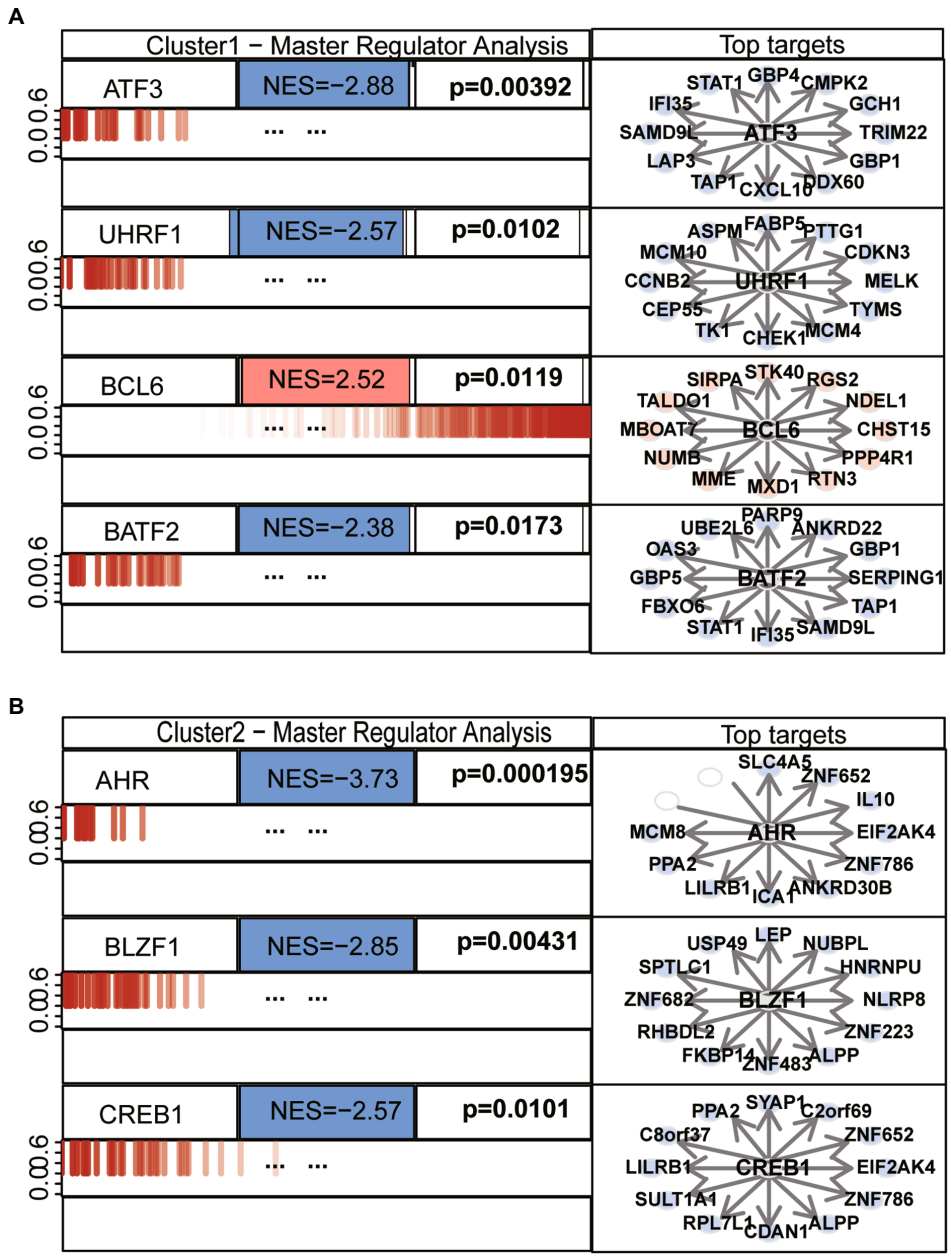
Top master regulators of ASD clusters. **(A)** Master regulatory analysis of ASD subcluster 1. **(B)** Master regulatory analysis of ASD subcluster 2. Each network is indicated by its master regulators. A barcode-like diagram displaying all transcriptome genes, from most downregulated (left) to most upregulated (right), is used to display the genes in each network (right). Targets with positive (red) and negative (blue) correlations are overlaid as bars of different colors on the differential expression signature. *p*-values and normalized enrichment scores (NES) are also shown. The 12 most likely network putative targets of each MR are shown to the right in red if they are upregulated or blue if they are downregulated, with a pointed arrow indicating that they are predicted to be activated by the centroid protein and a blunt arrow indicating that they are predicted to be repressed.

We performed differential analysis between ASD subclusters and obtained a total of 167 DEGs, of which 127 genes in ASD cluster 2 and 40 genes in ASD cluster 1 were expressed at lower levels (Figures 6A,B). Further enrichment analysis was carried out using these DEGs. GO enrichment analysis showed that ATP synthesis coupled electron transport, protein targeting to ER and phosphatidylinositol metabolic process were involved in the biological process subsection; Cellular component exhibits significant involvement of mitochondrial related complex in DEGs; Molecular function subsection data indicate the structural constituent of ribosome terms were most enriched (Figure 6C). Moreover, pathways of neurodegeneration-multiple diseases, oxidative phosphorylation, primary immunodeficiency interacted with the most number of genes according to the KEGG pathway database (Figure 6C). We also conducted the GSEA analysis, the results showed that REACTOME TRANSLATION (Figure 7A), REACTOME REGULATION OF EXPRESSION OF SLITS AND ROBOS (Figure 7B), REACTOME SELENOAMINO ACID METABOLISM (Figure 7C) and REACTOME RESPONSE OF EIF2AK4 GCN2 TO AMINO ACID DEFICIENCY (Figure 7D) pathway were significantly enriched in ASD cluster2, while KEGG LEISHMANIA INFECTION (Figure 7E), (Figure 7F), WP EBOLA VIRUS PATHWAY ON HOST (Figure 7G), KEGG SYSTEMIC LUPUS ERYTHEMATOSUS (Figure 7H) and PID FAS PATHWAY (Figure 7I) were significantly enriched in ASD cluster1. Next, we focused on the master regulator genes significant enriched in ASD clusters. The findings demonstrated that the transition from normal control to ASD cluster 1 was supported by the repression of ATF3, UHRF1, BATF2, and the activation of the BCL6 (Figure 8A). While inhibition of AHR, BZLF1, and CREB1 assisted the transition from normal control to ASD cluster 2 (Figure 8B). Overall, based on DEGs, functional enrichment and the master regulatory analysis, we highlighted two distinct biological phenotypes of ASD, which validated the existence of heterogeneity of ERS processes in ASD.

### 3.7. Identification of the immune microenvironment characteristics of different ER stress patterns

**Figure 9:**
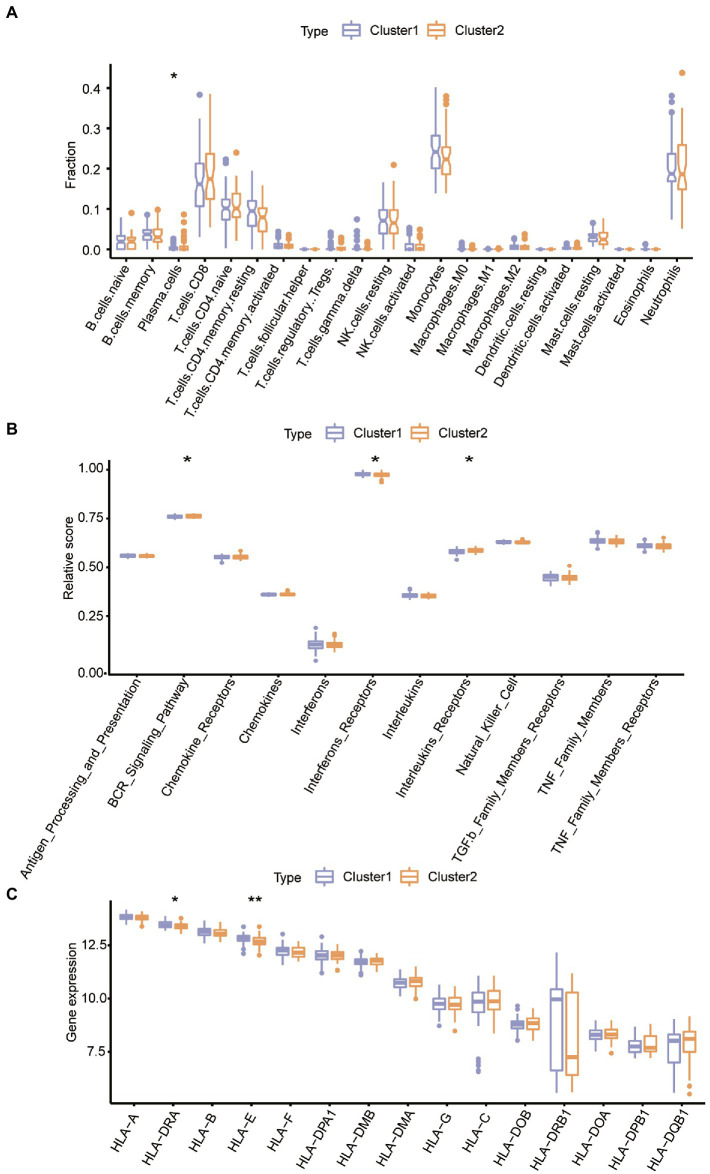
Different ER stress patterns are characterized by diversity in immune microenvironment features. **(A)** The variations of each immune microenvironment infiltrating immunocytes between ASD clusters. **(B)** The activity differences of each immune reaction gene set between ASD clusters. **(C)** The expression differences of each HLA gene between ASD clusters.

To examine the variations in the immune microenvironment, we compared the immune infiltrating cells, immune reaction gene sets and HLA gene expression between distinct subtypes. Analysis of the abundance of 22 immune microenvironment infiltrating cells revealed that ASD subtype 2 had a higher amount of plasma cell infiltration while the other 21 immune cells had a similar abundance (Figure 9A). Regarding the immune reaction process, the activity of the synonyms B cell receptor (BCR) signaling pathway and interleukin receptor activity were both increased in ASD subcluster2, but interferon receptor activity was decreased in subcluster2 (Figure 9B). Therefore, we speculated that a more active immune system in ASD subcluster2. In addition, the expression of different HLA was also different between ER stress patterns, HLA-DRA and HLA-E were downregulated in ASD subtype2 (Figure 9C). These results confirmed again that ER stress modification plays a crucial regulatory role in forming different immune microenvironments in ASD patients.

### 3.8. Identification of the potential compounds that target different ER stress patterns

**Figure 10:**
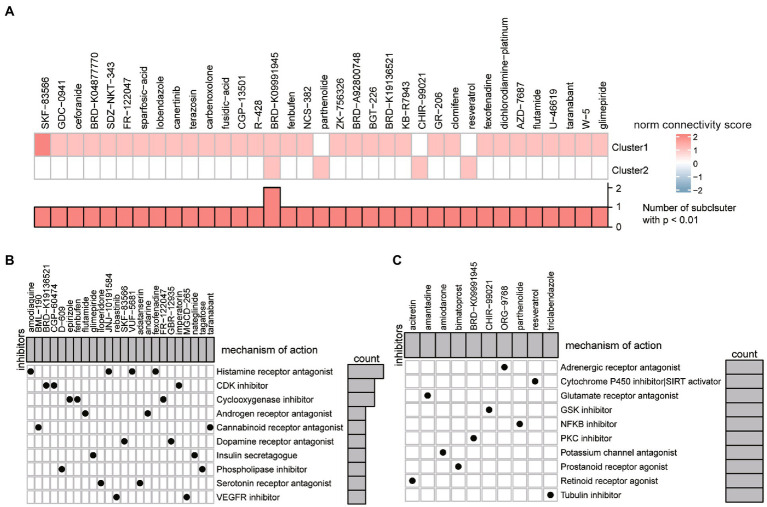
The potential compounds target different ER stress patterns. **(A)** Heatmap showing the scaled connectivity score of each compound for different ER stress patterns. **(B,C)** Heatmap displaying the mechanisms of action shared by prospective therapeutic compounds for cluster 1 **(B)** and cluster 2 **(C)**, respectively.

The CMap database was employed to find prospective compounds that can effectively reverse the differential gene expression of different ASD subclusters. Differences between Subcluster 1 and Subcluster 2 were also apparent in compound prediction (Figure 10A). We found a total of 118 compounds that were significantly enriched in the ASD subcluster1, and 18 compounds that were enriched in the ASD cluster2. Among them, the PKC inhibitor BRD-K09991945 that targets Glycogen synthase kinase 3β (GSK3B) might have a therapeutic effect on both clusters, whereas the PI3K inhibitor BGT-226 and Radical formation stimulant temoporfin might worsen the situation of different ASD subclusters, respectively (Supplementary Table S1). Furthermore, we conducted the MoA analysis of the potential compounds. The result of four compounds (fexofenadine, VUF-5681, JNJ-10191584 and amodiaquine) shared the MoA of histamine receptor antagonist, another three compounds (imperatorin, CGP-60474 and BRD-K19136521) shared the MoA of the CDK inhibitor in ASD subcluster1 (Figure 10B). Compounds that target ASD subcluster2 enriched the MoA of Adrenergic receptor antagonists, Cytochrome P450 inhibitor and other substances (Figure 10C).

## 4. Discussion

ASD are mental disabilities that have a serious impact on the quality of life of children with these disorders. Previous studies have shown that the activation of ER stress is associated with the occurrence and development of ASD (19, 21). The disruption of neuronal protein homeostasis (53–55), the dysregulation of calcium homeostasis (56, 57), the induction of inflammatory cytokines, such as TNF-α (58, 59), IL-33 (60), and IL-1β (61), and other pathways have all been proposed as contributing factors (59). Despite the previous role of ER stress in the pathophysiology of ASD has been highlighted, research on ER stress regulators in the ASD field remains in its infancy. To the best of our knowledge, this was the first in-depth bioinformatics analysis of the landscape of ER stress regulators in ASD disease, which may provide reliable directions for future experimental studies of ASD as well as novel chances to develop effective therapies.

In the present study, we first calculated the ER stress scores for each sample in GSE111176, and the results revealed that ER stress score was significantly higher in the ASD group; Differential expression analysis showed that there were 37 ER stress regulators altered in the ASD patients, suggesting that ER stress might play a key role in the ASD that deserves further investigation.

Parental interviews and the use of scales to score the specific symptoms displayed by the affected kid are the current diagnostic techniques for ASD, but they are both subjective. Recently, we are starting to see the impact of machine learning methods on data modeling and classification. Using random forest and artificial neuron network methods, a classifier built on the seven ER stress regulators could be effective at differentiating between healthy people and ASD patients (AUC >0.7 among independent datasets), which may help a clinician diagnose in the future.

Moreover, WGCNA is a powerful tool that provides module construction and correlation analysis within the gene expression data to determine the associations between modules and pathological features of the disease. We investigated the co-expression modules linked to elevated ER stress scores in ASD patients using the WGCNA, and the turquoise module with 774 genes showed a significant correlation with ER stress scores. To increase the reliability of the data, we took the intersection of turquoise modules and differentially expressed ER stress regulators, referring to them as hub ASD-related ER stress genes, including SEC16A, EXTL3, DNAJB14, SGTA, and UMF1. SEC16A gene encodes a protein that forms part of the Sec16 complex, which is involved in protein transport from the endoplasmic reticulum (ER) to the Golgi and mediates COPII vesicle formation at the transitional ER. Jing-Jing Sun and colleagues found that SEC16A mRNA expression was higher in ASD serum samples, and it was able to form a diagnostic model with four additional genes to aid in the diagnosis of ASD (62). UFM1 is a ubiquitin-like protein that is conjugated to target proteins by the E1-like activating enzyme UBA5 and E2-like conjugating enzyme UFC1 in a manner analogous to ubiquitylation. Individuals with UBA5 variants can develop severe irritability, stagnation of development and epilepsy (63). However, the association between the aforementioned genes and ASD is still unknown, future studies can include functional studies to elucidate such relationships.

Recently, based on the inter-individual deviation of functional connectivity (IDFC), Xiaonan Guo and colleagues found two ASD subtypes, each with a different level of social communication deficits and confined and repetitive behaviors (64). Ada J S Chan and his colleagues discovered that distinct genetic subtypes of ASD can enable the prediction of developmental outcomes (65). Molecular subtyping strategy is widely utilized in the biomedicine field, and the identification of novel molecular subtypes may lead to a better treatment plan. In this study, ER stress patterns in ASD were explored using hub ER stress regulator expression profiles and unsupervised clustering analysis, and two subclusters with distinct ER stress patterns were discovered. Understanding the distinctions in their biological roles may aid in understanding the involvement of ER stress in ASD etiology. A meta-analysis revealed that genes in the FAS signaling were upregulated in the ASD (66)， however, we found that the FAS pathway was more enriched in the subcluster1 than subcluster2. This further demonstrates that molecular typing is more conducive to the precise treatment of patients. Aside from transcript differences, each subcluster has particular immunological traits of its own. The ASD subcluster2 has a higher infiltration level of plasma cells and higher activity of the BCR signaling pathway and interleukin receptor reaction. Previous research has shown the role of both adaptive and innate immune cells in the etiology of ASD. It was reported that a single injection of IL-6 into pregnant mice led to autism-relevant behaviors in the offspring, while blockade of IL-6 trans-signaling in the brain of mice could cause improved autism-like behavioral symptoms (56, 57). Ahmed Nadeem and colleagues revealed that dysregulation in IL-6 receptors is associated with upregulated IL-17A-related signaling in children with autism, and the IL-6/IL-17A-related parameters are positively correlated with disease severity (58). The immunological traits of each subcluster confirmed the accuracy of the classification of various ER stress regulators utilized in this investigation.

At this time, anti-psychotic medicines like aripiprazole and risperidone continue to be the mainstay of ASD pharmacological treatment. However, due to its metabolic and neurological effects, long-term efficacy and safety concerns continue to be debatable. More studies are still required to develop more effective and targeted interventional treatments. After clarifying that different ASD subclusters have diverse expression patterns and immunological features, we attempted to anticipate compounds for different subclusters to achieve accurate treatment. The findings demonstrated that the compound prediction outcomes for subtypes 1 and 2 were highly different, with more compounds targeting subcluster 1 than subcluster 2, suggesting a greater emphasis on a comprehensive assessment of the patients. ASD subclusters could be studied further at the molecular or immune level rather than the phenotypic level. We found the PKC inhibitor BRD-K09991945 that targets GSK3B might have a therapeutic effect on both clusters. In the previous study, the p-GSK-3β (S9) was increased in the ASD mice model, and the low-frequency rTMS (LF-rTMS) was able to lower the phosphorylation level of the GSK-3β and so improve the social function of mice (67). Therefore, the PKC inhibitor BRD-K09991945 can be considered for further verification by animal experiments or clinical trials.

## 5. Conclusion

In conclusion, our study revealed that ER stress score was significantly higher in ASD patients. The classifier consists of the seven ER stress regulators that can accurately predict the prevalence of ASD. Furthermore, we identified two ER stress patterns and found that the diversity of ER stress patterns affects the heterogeneity and complexity of the immune microenvironment in ASD. The comprehensive analysis of the ASD ER stress patterns will make a great contribution to understanding the underlying pathophysiology of ASD, inspiring more effective therapeutic approaches.

## Data availability statement

Publicly available datasets were analyzed in this study. This data can be found here: https://www.ncbi.nlm.nih.gov/geo/ with accession numbers GSE111176, GSE77103 and GSE3832.

## Author contributions

YM conceived and designed the study. YL developed the methodology. SG analyzed and interpreted the data. YL, YM, and SG wrote and revised the manuscript. All authors contributed to the article and approved the submitted version.

## Conflict of interest

The authors declare that the research was conducted in the absence of any commercial or financial relationships that could be construed as a potential conflict of interest.

## Publisher’s note

All claims expressed in this article are solely those of the authors and do not necessarily represent those of their affiliated organizations, or those of the publisher, the editors and the reviewers. Any product that may be evaluated in this article, or claim that may be made by its manufacturer, is not guaranteed or endorsed by the publisher.

## Footnotes

^1^ http://www.immport.org

^2^ https://www.networkanalyst.ca/
