# Supplementary figures and images for: Integrated analysis of endoplasmic reticulum stress regulators’ expression identifies distinct subtypes of autism spectrum disorder
Source: Front Psychiatry. 2023 Apr 17;14:1136154. doi: 10.3389/fpsyt.2023.1136154 (PMC10149679; doi:10.3389/fpsyt.2023.1136154)

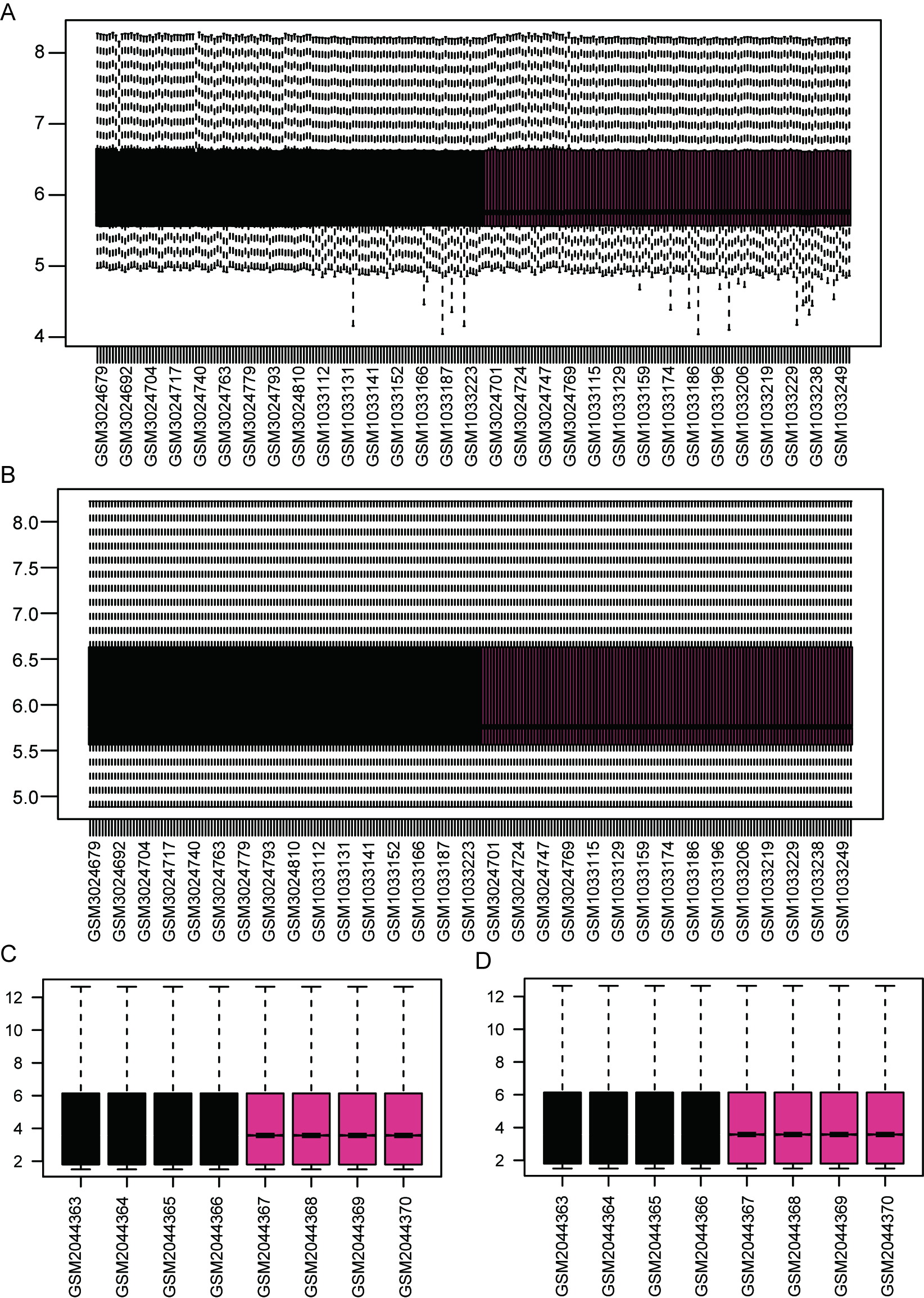

Supplement: Supplementary file 1 [file Image_1.TIF]
